# P-1965. Non-Pharmaceutical Interventions and Protection Against SARS-CoV-2 Infection in a Changing Pandemic Landscape

**DOI:** 10.1093/ofid/ofae631.2124

**Published:** 2025-01-29

**Authors:** Meri Varkila, Catherine Ley, Maria de la Luz Sanchez, Julie Parsonnet

**Affiliations:** Stanford University, Stanford, California; Stanford University, Stanford, California; Stanford University, Stanford, California; Stanford School of Medicine, Stanford, California

## Abstract

**Background:**

Non-pharmaceutical interventions (NPI), such as social distancing measures and the use of face masks, are presumed to lower the transmission of SARS-CoV-2. Guidance and mandates by public health authorities, however, have varied throughout the COVID-19 pandemic. This study sought to assess, on a population level, whether self-reported NPI measures were associated with the risk of SARS-CoV-2 infection at different times in the pandemic.

**Figure 1.**
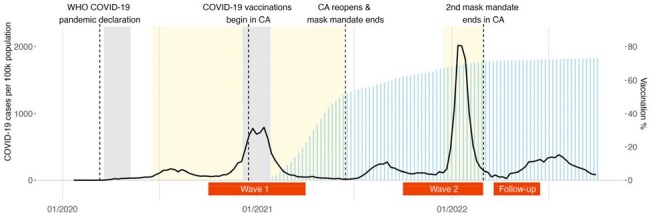
Study data collection in relation to COVID-19 cases and pandemic milestone events in California in 2020-2022.

The black solid line depicts newly reported COVID-19 cases in California across time. The grey shaded areas show the duration of the first and second shelter-in-place orders in California. The yellow shaded areas show the duration of the regional mask mandates in California. The light blue bars illustrate the cumulative weekly vaccination percentage across time. The red horizontal bars depict when CA-FACTS surveys were conducted. WHO; World Health Organization, CA; California.

**Methods:**

We conducted a repeated cross-sectional study among randomly sampled households in 3 Northern California counties over 3 time points: Oct 2020-Mar 2021 (wave 1), Oct 2021-Feb 2022 (wave 2) and Mar-Jun 2022 (follow-up survey of wave 2 participants) (Figure 1). Social distancing, face mask use, vaccination status and self-reported COVID-19 were recorded by participants’ response in an online survey. We describe changes in NPI adoption across time and estimate the association of NPI and risk of COVID-19 infection among vaccinated individuals using logistic regression.

**Figure:**
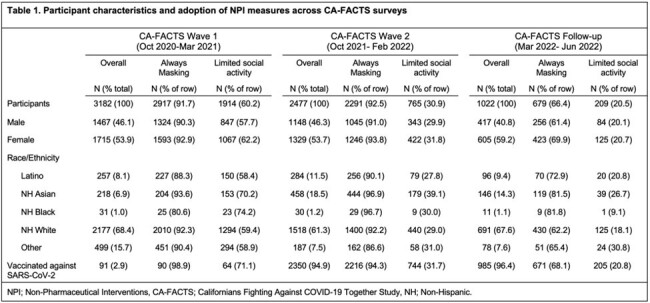

**Results:**

The proportion of participants who reported never or rarely participating in social activities outside their household decreased from 60.2% in wave 1 to 20.5% in the follow-up survey. Similarly, face mask use decreased over time with 91.7% of wave 1 and 92.5% of wave 2 participants reporting consistently wearing a face mask in indoor public spaces compared to 66.4% of follow-up participants (Table 1). In multivariable regression models, social distancing was associated with significantly reduced odds of COVID-19 among vaccinated individuals in both surveys (wave 2 aOR 0.53, 95%CI 0.33-0.81; follow-up aOR 0.20, 95%CI 0.08-0.40). Consistent use of face masks tended to be less in those with self-reported COVID-19 in wave 2 (adjusted odds ratio (aOR) 0.58, 95%CI 0.32-1.13) but showed little impact in the follow-up survey (aOR 0.96, 95%CI 0.65-1.44, Table 2).

**Figure:**
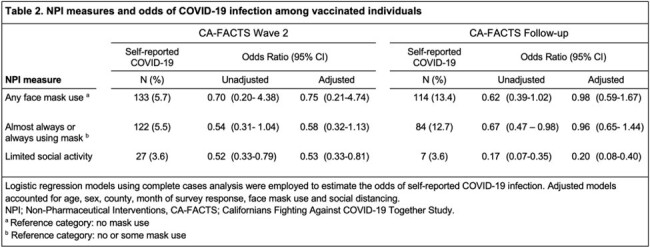

**Conclusion:**

Declines in protection offered by face masking coincided with the lifting of mask mandates and decreased adherence to face mask use in communities overall. In contrast, the importance of social distancing as a protective strategy against COVID-19 infection appeared to increase over time. NPI, particularly social distancing, should remain first-line approaches to preventing infection in future outbreaks.

**Disclosures:**

All Authors: No reported disclosures

